# Selection and characterization of a SpaCBA pilus-secreting food-grade derivative of *Lacticaseibacillus rhamnosus* GG

**DOI:** 10.1007/s00253-020-11051-7

**Published:** 2021-01-08

**Authors:** Hanne L. P. Tytgat, Pia Rasinkangas, Jarmo Ritari, Justus Reunanen, Steven Aalvink, Chia-wei Lin, Airi Palva, François P. Douillard, Willem M. de Vos

**Affiliations:** 1 Laboratory of Microbiology, Wageningen University, Wageningen, The Netherlands; 2 Department of Veterinary Biosciences, Faculty of Veterinary Medicine, University of Helsinki, Helsinki, Finland; 3 DuPont Nutrition & Biosciences, Kantvik, Finland; 4 Finnish Red Cross Blood Service, Helsinki, Finland; 5 University of Oulu, Oulu, Finland; 6 Functional Genomics Center Zurich, ETH Zurich, Zurich, Switzerland; 7 Present Address: Department of Food Hygiene and Environmental Health, Faculty of Veterinary Medicine, University of Helsinki, Helsinki, Finland; 8 Human Microbiome Research Program, Faculty of Medicine, University of Helsinki, Helsinki, Finland

**Keywords:** Pili, Non-GMO derivative, Pilus biogenesis, *L. rhamnosus* GG

## Abstract

Many studies have established the functional properties of *Lacticaseibacillus rhamnosus* GG, previously known as *Lactobacillus rhamnosus* GG, marketed worldwide as a probiotic. The extraordinary capacity of *L. rhamnosus* GG to bind to human mucus and influence the immune system especially stand out. Earlier, we have shown the key role of its SpaCBA sortase-dependent pili encoded by the *spaCBA-srtC1* gene cluster herein. These heterotrimeric pili consist of a shaft pilin SpaA, a basal pilin SpaB, and tip pilin SpaC that contains a mucus-binding domain. Here, we set out to characterize a food-grade non-GMO mutant of *L. rhamnosus* GG, strain PA11, which secretes its pilins, rather than coupling them to the cell surface, due to a defect in the housekeeping sortase A. The sortase-negative strain PA11 was extensively characterized using functional genomics and biochemical approaches and found to secrete the SpaCBA pili into the supernatant. Given the functional importance and uniqueness of the mucus-binding pili of *L. rhamnosus* GG, strain PA11 offers novel opportunities towards the characterization and further therapeutic application of SpaCBA pili and their low-cost, large-scale production.

*•Creation of pilus-secreting mutant (PA11) of the key probiotic LGG.*

*•Strain PA11 is defective in a functional housekeeping sortase SrtA.*

*•Strain PA11 opens novel biotherapeutic application avenues.*

Graphical abstract

**Supplementary Information:**

The online version contains supplementary material available at 10.1007/s00253-020-11051-7.

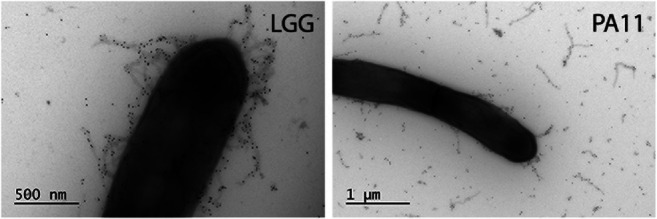

## Introduction

*Lacticaseibacillus rhamnosus* GG (previously known as *Lactobacillus rhamnosus* GG) (Zheng et al. 2020) is a widely marketed lactic acid bacterium (commercialized as LGG^®^) that has been extensively studied for its probiotic properties for decades (Saxelin et al. 2005). Among others, the beneficial effect of *L. rhamnosus* GG has been demonstrated in the prevention of diarrhea in children and adults (Arvola et al. 1999; Wolvers et al. 2010; Szajewska and Kolodziej 2015) and respiratory tract infections in children (Hatakka et al. 2001; Kumpu et al. 2012; Luoto et al. 2014). This effect has been mainly associated to the high adhesion capacity of *L. rhamnosus* GG attributed to the presence of unique mucus-binding pili on its surface, which are absent in closely related strains, such as *L. rhamnosus* LC705 (Kankainen et al. 2009). These sortase-dependent pili are heterotrimeric proteinaceous cell wall appendages involved in adherence, biofilm formation, and host signaling (Kankainen et al. 2009; von Ossowski et al. 2010; Lebeer et al. 2012; Ardita et al. 2014; Chandrasekharan et al. 2019).

The biogenesis of the *L. rhamnosus* GG pili is encoded by the *spaCBA-srtC1* gene cluster (Kankainen et al. 2009). The pilus shaft is built out of the major pilin SpaA, while the SpaB pilin terminates pilus synthesis (Reunanen et al. 2012; Douillard et al. 2014). The pilus shaft and tip are decorated with a mucin-binding domain containing SpaC subunit (Reunanen et al. 2012). The latter subunit is the major contributor to the strong mucus adhesion phenotype of *L. rhamnosus* GG (Kankainen et al. 2009) by specifically binding to the non-reducing termini of mucus glycoconjugates (Nishiyama et al. 2016). More in particular, the SpaC pilin harbors a C-terminal collagen-binding domain and an N-terminal von Willebrand factor type A domain (Tripathi et al. 2013). Especially, the latter one is thought to contribute to the extraordinary adhesion capacities of the SpaC pilin and by extension of the SpaCBA pili and *L. rhamnosus* GG cells (Tripathi et al. 2013; Kant et al. 2016). The different pilin subunits are polymerized to form the pilus, with lengths of over 1 μm by a pilin-specific transpeptidase sortase SrtC1, which recognizes highly conserved LPxTG and YPKN motifs and covalently attached to the cell wall by a housekeeping sortase, SrtA (Hendrickx et al. 2011; Reunanen et al. 2012). The action of both sortases during assembly and anchoring of the pilus is further determined by either a triple or single glycine motif recognized by the pilin-specific sortase and housekeeping sortase of *L. rhamnosus* GG, respectively (Douillard et al. 2014). Further investigation of the pilin-specific SrtC1 revealed an N-terminal GYPSY domain important for its functionality (Douillard et al. 2016). The intensive study of these peculiar pili of *L. rhamnosus* GG has already generated important insights in pilus assembly and function in Gram-positive species in general. Sortase-dependent pili are key cell wall appendages of Gram-positive species, especially in pathogens, where they are virulence factors (Mandlik et al. 2008). In earlier work, we uncovered a striking structural and functional resemblance between the pili of the probiotic *L. rhamnosus* GG and the nosocomial pathogen Vancomycin-resistant *Enterococcus* (VRE), showing that SpaCBA pili can drive competitive exclusion of VRE (Tytgat et al. 2016a).

To get insight into the genes governing SpaCBA pilus formation and to produce a panel of strains that can be safely used in therapeutic applications, we earlier set out to produce and characterize non-piliated derivatives of *L. rhamnosus* GG using a chemical mutagenesis approach (Rasinkangas et al. 2014). Since random chemical mutagenesis was used to produce these, the resulting *L. rhamnosus* GG derivatives are allowed to be used in human trials, as they are not considered to be genetically modified organisms (GMO) and can be used in food applications in the EU, that is known for its strict regulation (EU 2001). In this food-grade mutagenesis campaign, we also obtained strain PA11 from *L. rhamnosus* GG that contains a critical mutation in the house-keeping sortase (*srtA*) gene and was found to secrete the mucus-binding SpaC-harboring pili. In this work, we report on the in-depth characterization of *L. rhamnosus* PA11 at the phenotypic, biochemical, and genomic level.

## Materials and methods

### Bacterial strains and growth conditions

*Lacticaseibacillus rhamnosus* GG (ATCC 53103) and *Lacticaseibacillus rhamnosus* LC705 were obtained from Valio Ltd., Helsinki, Finland. The previously characterized *L. rhamnosus* GG PB12 strain (Rasinkangas et al. 2014) and closely related *L. rhamnosus* LC705 were included as SpaCBA pilus-less controls in experiments. All strains were grown in De Man-Rogosa-Sharpe (MRS) medium and agar (Difco) at 37 °C.

### Mutagenesis

Mutagenesis was performed as described earlier in Rasinkangas et al. (Rasinkangas et al. 2014) using 2% (*v*/*v*) ethyl methanesulphonate (EMS; Sigma Aldrich). EMS is an alkylating mutagen and produces mainly transition mutations (Sega 1984; Parekh et al. 2000). The *L. rhamnosus* GG derivative strain PA11 was isolated based on a previously described enrichment procedure where we selected for mutants that were reduced in the binding to mucus in microtiter plates (Rasinkangas et al. 2014). Strain PA11 was obtained among a dozen of pilus-less mutants and stood out because it did not bind mucus, but still produced pili. *L. rhamnosus* PA11 is available under number DSM 111733 at the DSM-Z Braunschweig, Germany.

### Immunoelectron microscopy

Immunoelectron microscopy double labeling was performed as described previously (Reunanen et al. 2012; Rasinkangas et al. 2014), with antisera against SpaA and SpaC pilins (Douillard et al. 2016; Tytgat et al. 2016b). SpaA was labeled with 10 nm, and SpaC was labeled with 5 nm protein A-gold particles. The samples were visualized using JEM-1400 transmission electron microscope (JEOL).

### Quantification of surface-located pilins using immunofluorescence

Immunofluorescence labeling, quantification, and microscopy of pilins on the surface of cells were performed essentially as described previously in Rasinkangas et al. (2014). Initial labeling was performed using SpaA and SpaC antisera followed by secondary labeling with Alexa Fluor 488-labeled goat anti-rabbit antibody (Invitrogen). To normalize the fluorescence intensity for SpaA and SpaC to the total quantity of cells in the samples, chromosomal DNA of the cells was labeled with 1:1000-diluted 4′,6-diamidino-2-phenylindole (DAPI, Dilactate-form, Thermo Fisher Scientific) at the same time with the secondary antibody labeling. Fluorescence intensities of freshly labeled samples were measured with a Victor3 1460 multilabel counter (Perkin Elmer) using separate measurement programs for each dye. The fluorescence intensity results from the immunofluorescence labeling were divided by the corresponding DAPI intensity to obtain a normalized fluorescence value for each strain.

### Mucus adherence measurements

Mucus adherence of the strains was measured as described previously (Vesterlund et al. 2005; Rasinkangas et al. 2014). In short, strains were grown overnight in the presence of ^3^H-labeled thymidine (Perkin Elmer). These cultures were then incubated in a Maxisorp microtiter plate coated with porcine type II mucus (Sigma). Wells were washed with PBS (Oxoid) and the remaining adherent cells were lysed. Radioactivity of the samples was measured with a Wallac 1480 Wizard 3 automatic gamma counter (Perkin Elmer). Three repeats of the mucus binding assay were performed, and in each assay, 3–6 technical replicates were prepared for each strain. Data analysis was performed using GraphPad Prism® 8. Significant differences between two groups were calculated using unpaired *t* tests, and the significance level was set at *p* < 0.0001.

### Genome sequencing

Extraction of genomic DNA, shotgun genome sequencing (Illumina, MiSeq), and processing of sequences were performed as described earlier (Rasinkangas et al. 2014). Sequence assemblies were aligned to the *L. rhamnosus* GG genome (Kankainen et al. 2009) using MUMmer 3.0 software (Kurtz et al. 2004) as described previously. The mutation detected in the *srtA* gene of the PA11 strain was further verified with Sanger sequencing. Genomic DNA was also sent to BaseClear (Leiden, NL), where single-molecule genome sequencing (PacBio) was performed and analyzed according to their procedures (paired-end sequencing on Illumina HiSeq2500 system followed by variant detection).

### Western blot analysis of the cell wall-located pilins

Cell wall proteins were extracted as detailed previously (Avall-Jaaskelainen et al. 2003; Rasinkangas et al. 2014) and analyzed with Western blot, as described in Rasinkangas et al. (Rasinkangas et al. 2014). The optical density of the cultures was adjusted to *A*_600_ = 8.0 before protein extraction, and the protein extracts were diluted 1:300 for detection with SpaA antiserum and 1:200 for SpaC Western blot. Secreted proteins were analyzed by blotting a 10.5-μl sample from the culture medium.

### Secretome

The supernatant of a 500-ml culture of *L. rhamnosus* GG wild type and PA11 at OD_600_ 7 and was collected and filtered over a 0.45-μm filter. After TCA precipitation (20% (*w*/*v*) final concentration, Sigma), the pellet was washed twice with ice-cold acetone. Dried samples were resuspended in 200 uL 8% (w/v) SDS and further in 400 uL ddH_2_O, using sonication in a water bath to dissolve the samples. Samples were run on an 8% SDS gel after which the whole lane was cut into ten pieces and prepared for further analysis by nanoUPLC-MS/MS. The gel pieces were processed as described earlier (Lee et al. 2003). In brief, the gels were reduced and alkylated by resp. 10 mM dithiothreitol and 20 mM iodoacetamide. The gel pieces were washed twice in 50% (*v*/*v*) acetonitrile in 50 mM ammonium bicarbonate buffer pH 8.5, followed by a wash with 100% (*v*/*v*) acetonitrile. Gel pieces were dried by SpeedVac and incubated with 0.1 μg of trypsin overnight. Tryptic peptides were desalted by C18 Zip-Tip (Millipore, USA) and dried by SpeedVac. Twenty percent of the dissolved peptide sample was analyzed using an Easy-nLC™ 1000 system (Thermo Scientific) coupled to a LTQ-Orbitrap Fusion mass spectrometer (Thermo Scientific). Peptides were resuspended in 2.5% (*v*/*v*) acetonitrile (ACN) with 0.1% (*v*/*v*) formic acid (FA) and loaded on a self-made fritted column (75 μm × 150 mm) packed with reverse phase C18 material AQ, 3 μm 200 Å, (Bischoff GmbH, Leonberg, Germany) and eluted by a gradient (from 5 to 35% of solution B (99.9% ACN, 0.1% FA) for 30 min, 55% of B for 10 min, 97% of B for 10 min, with a flow rate of 300 nl/min). One scan cycle included a full-scan MS survey spectrum, followed by sequential HCD MS/MS on the most intense signals (> 50,000) to the maximal cycle time for 3 s. Full-scan MS spectra (400–2000 *m*/*z*) and HCD MS/MS spectra were recorded in the FT-Orbitrap (resolution of 120,000 at 400 *m*/*z* for MS and 60,000 at 400 *m*/*z* for MS/MS). HCD was performed with a target value of 5e4 and stepped collision energy rolling from 35 NCE was applied. For full Fourier transform MS, AGC target values were 2e5. Dynamic exclusion with a single repeat count, 15 s repeat duration, and 60 s exclusion duration was used for all experiments. The data was transformed to mgf file format by Proteome Discoverer 2.0 (Thermo, USA) and searched against the *L. rhamnosus* GG proteome (Kankainen et al. 2009) using the Mascot search engine. Quantification was performed by Progenesis QI.

## Results

Following up on our previous work selecting and characterizing non-mucus binding non-GMO derivatives of the model probiotic *L. rhamnosus* GG, we report on the isolation and characterization of the PA11 derivative that was found to secrete its SpaCBA pili.

### Selection of pili-secreting *L. rhamnosus* GG derivative

Using ethyl methanesulfonate (EMS) as a chemical mutagenesis agent, derivatives of *L. rhamnosus* GG (ATCC 53103) were generated and non-anti-SpaC-binding mutants were enriched and further characterized (Rasinkangas et al. 2014). One isolate, strain PA11, in particular, stood out during the characterization of the non-mucus adherent strains as it appeared to secrete its pilins, which triggered a more in-depth investigation.

### Immunoelectron microscopy shows that PA11 secretes its pili

**Fig. 1:**
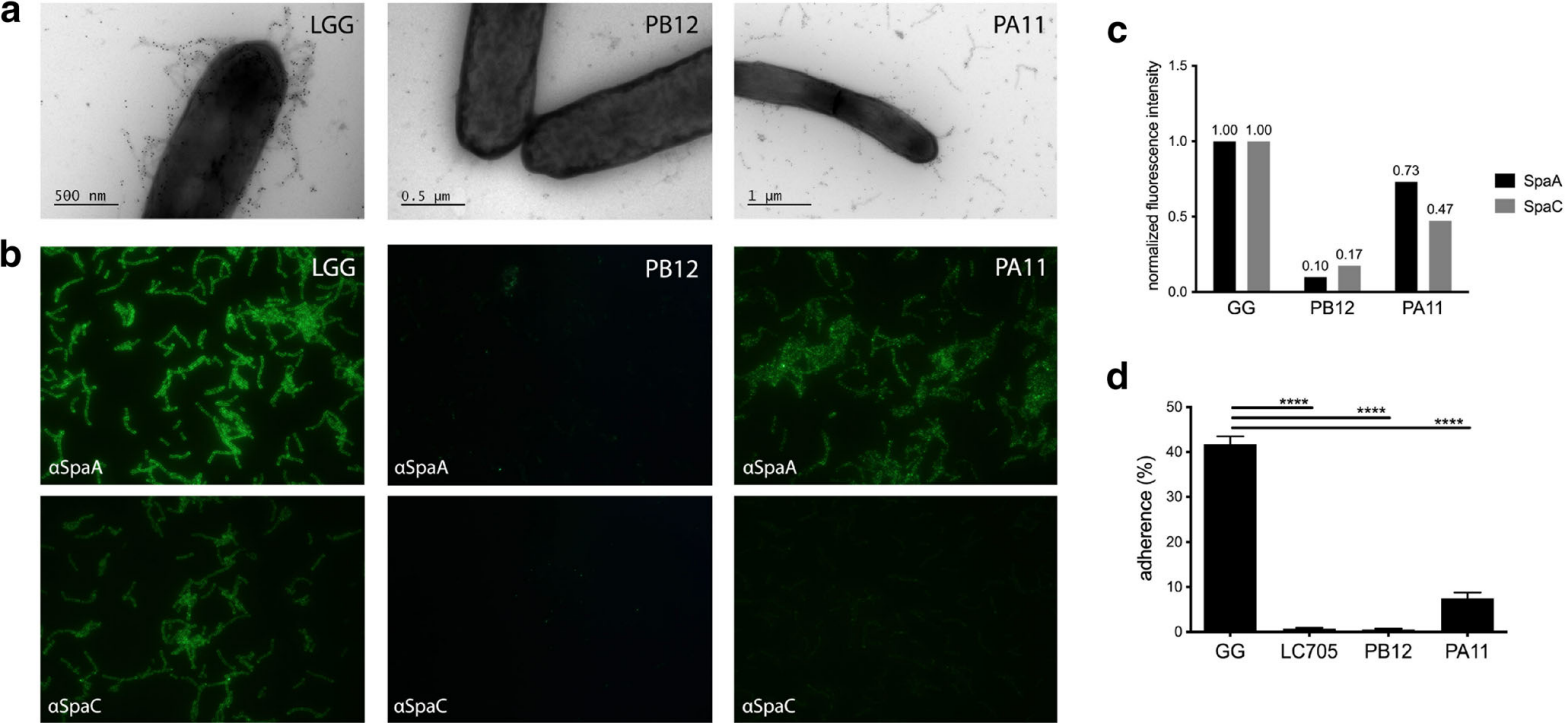
Phenotypical characterization of the pilus-secreting *L. rhamnosus* GG derivative PA11. **a** Immunoelectron micrographs of SpaC-SpaA-double labeling of *L. rhamnosus* strains. SpaC is labeled with 5 nm gold particles, while SpaA is labeled with 10 nm gold particles. A representative figure is shown for *L. rhamnosus* GG, the pilus-deficient PB12, and the pilus-secreting derivative PA11. **b** SpaA and SpaC immunofluorescence labeling. The strains were labeled with SpaA and SpaC antisera, and the antibodies were detected with Alexa 488-labeled secondary antibody. **c** SpaA and SpaC immunofluorescence labeling quantitative results. DAPI-normalized fluorescence intensity measurement results. Results are normalized to values for the parental strain both for SpaA and SpaC quantification. A representative experiment is shown. **d** Adherence of the different strains to porcine mucus. Wild-type *L. rhamnosus* GG was included as positive control, while the closely related, but SpaCBA-negative strain, *L. rhamnosus* LC705 and the non-piliated derivative PB12 were included as negative controls. The result is the average of 3 biological measurements, in which 3–6 technical repeats were included. Standard deviation is depicted

We used immunoelectron microscopy (immuno-EM) to reveal if and how the pili of strain PA11 are displayed on the cells. Cells were treated both with 10 nm gold particles coupled with anti-SpaA (shaft pilin) antibodies and 5 nm gold particles coupled to anti-SpaC (tip pilin) antibodies. Labeling of the wild-type strain showed co-labeling of both particles on the proteinaceous appendages on the cell surface, while labeling was absent in the non-piliated PB12 strain we described earlier (Fig. 1a) (Rasinkangas et al. 2014). The cells of strain PA11 were practically non-piliated, only showing minor amounts of pili on the cell surface. In contrast to the picture of strain PB12, the bulk of the pili of strain PA11 appeared to be loosely secreted in the milieu surrounding the cells, as evident by the interaction of the gold particles with these secreted pili (Fig. 1a).

### Quantification of pilins using immunofluorescence labeling

In order to confirm the immuno-EM observations and quantify the amount of pili produced by each strain, we performed immunofluorescence assays. Fluorescence of each sample was normalized to DAPI staining of nucleic acids, and all values were compared to the wild-type strain. In line with the immuno-EM observations, the quantity of SpaA and SpaC pilins at the surface of the pilus-secreting PA11 strain was lower compared to the parental strain (with a normalized fluorescence intensity for SpaA of 0.73, and for SpaC of 0.47), albeit slightly higher than in the pilus-deficient strain PB12 (SpaA 0.1, SpaC 0.2) (Fig. 1b, c). The apparent absence of SpaC staining of strain PA11 is in accordance with its selection process, as mutants were selected based on their non-interaction with anti-SpaC antibodies (Fig. 1b) (Rasinkangas et al. 2014).

### Strain PA11 is deficient in mucus adhesion

Given the microscopical observation suggesting that strain PA11 secretes its pili, a mucus adhesion experiment was set up to investigate how this affected the ability of PA11 to interact with mucus. Mucus adhesion to porcine mucus was significantly reduced (*p* < 0.0001) in the pilus-secreting derivative PA11 (6.4% of the cells, SEM 0.6) compared to the parental strain (41.7%, SEM 1.7) (Fig. 1d). The pilus-less strain PB12 and the closely related *L. rhamnosus* LC705, which is deficient in the *spaCBA-srtC1* genes (Kankainen et al. 2009), were included as negative controls (respectively 0.58%, SEM 0.18 and 0.77%, SEM 0.15). Our results further corroborate the importance of SpaCBA pili in the strong mucus adherence phenotype of *L. rhamnosus* GG (Kankainen et al. 2009; von Ossowski et al. 2010; Lebeer et al. 2012).

### Genome analysis reveals a mutation in the housekeeping sortase SrtA

**Fig. 2:**
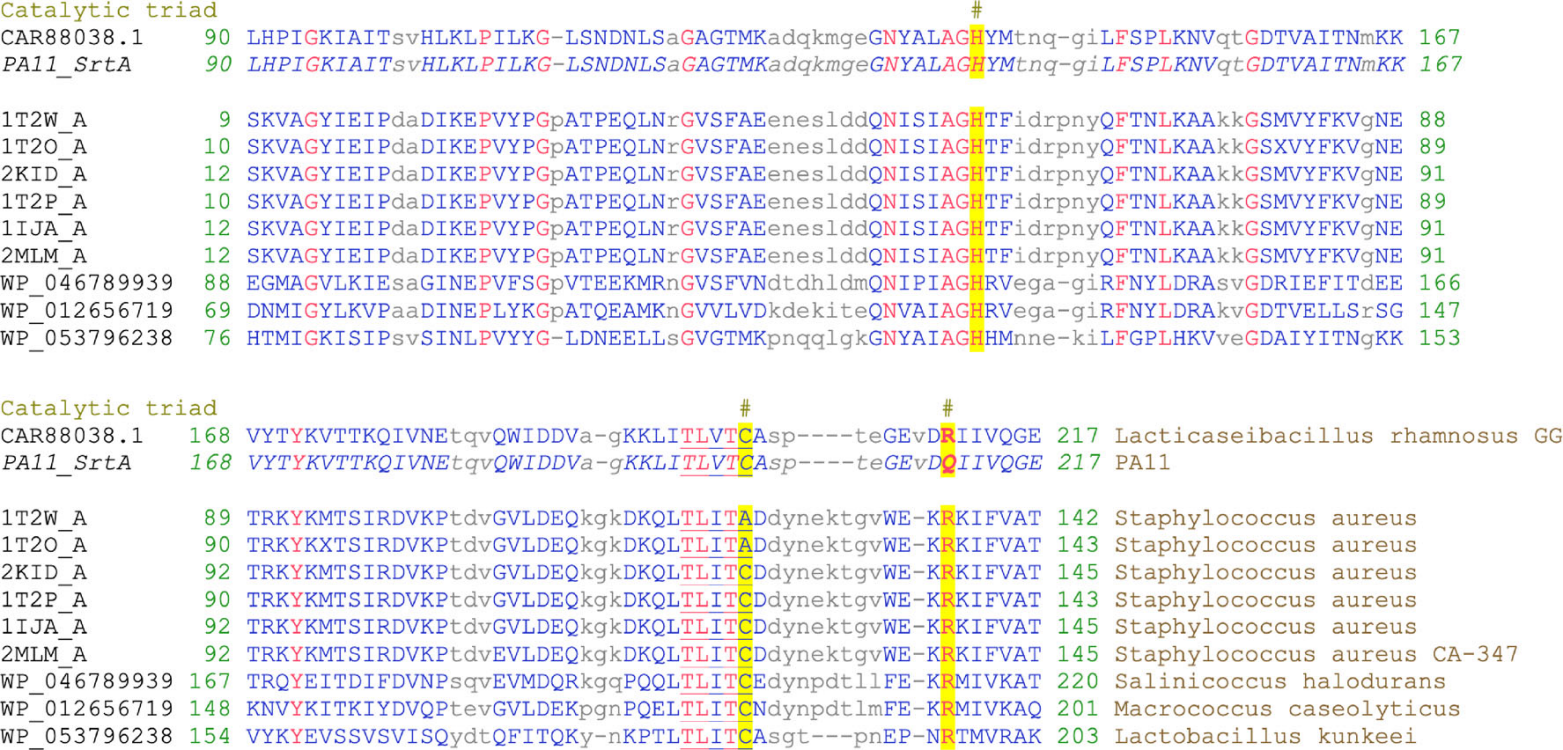
Multiple sequence alignment of SrtA sequences. The protein sequence of *L. rhamnosus* GG SrtA was aligned against members of the SrtA protein family, cd06165. The catalytic triad is indicated with a number sign, and the TXTLC catalytic motif is underlined. The mutation of residue 211 in the PA11 strain of an Arg into a Gln, resulted in the substitution of one of the three amino acids of the catalytic triad: His, Cys, and Arg

To investigate the genotype underlying our phenotypic observations on the secretion of pili into the growth medium by strain PA11, we performed deep shotgun (Illumina MiSeq, 142-fold coverage, 130 Mbp, Rasinkangas et al. 2014) as well as single-molecule (PacBio, BaseClear, Leiden, NL) sequencing of its genome (Supplementary File 1). The genome sequence of *L. rhamnosus* GG-derived strain PA11 can be found in the NCBI Sequence Read Archive (SRA) database with accession number SAMN04440354, and all sequencing data was deposited as part of BioProject PRJNA309744. In contrast to the earlier characterized non-piliated derivatives of *L. rhamnosus* GG (Rasinkangas et al. 2014), the *spaCBA-srtC1* gene cluster responsible for SpaCBA pili production was not affected. A total of 86 SNPs were detected with shotgun sequencing and 103 SNPs in 87 genes with single-molecule sequencing, including a SNP in the *srtA* gene encoding the housekeeping sortase SrtA, *LGG_RS10305* (*LGG_02143*) (Supplementary File 1). The mutation detected in the *srtA* gene of the PA11 strain was further verified with Sanger sequencing and included a point mutation changing the G at position 632 to an A, resulting in a change of amino acid residue at position 211 from Arg to Gln (Supplementary File 1; Fig. 2).

### Western blot analysis points towards secretion of mature pili by PA11

**Fig. 3:**
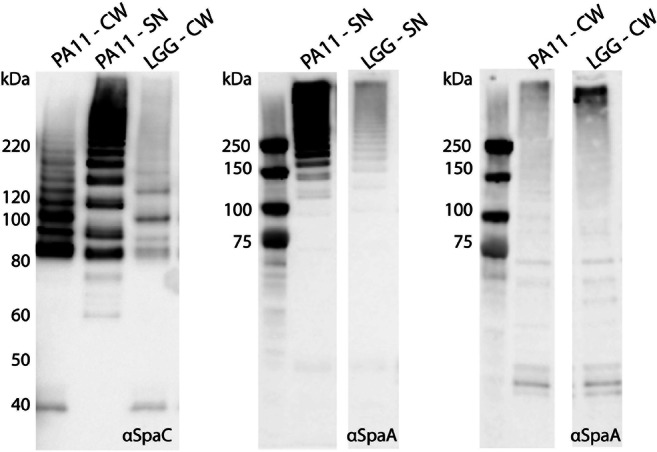
Western blot analyses of PA11 supernatant and cell wall protein fractions. Both SpaA and SpaC blots are shown for cell wall (CW) and supernatant (SN) protein fractions of *L. rhamnosus* GG and PA11. The cell wall extracts were extracted as detailed previously (Avall-Jaaskelainen et al. 2003; Rasinkangas et al. 2014) from cultures with an adjusted optical density of *A*_600_ = 8.0. Samples were diluted 1:300 for the SpaA and 1:200 for the SpaC blot. To analyze proteins secreted by both strains, a 10-μl aliquot of the cell culture medium was blotted

Based on the genotypical and phenotypical results described above, we set out to further strengthen the observation that strain PA11 secretes most of its mature pilins into its environment. Cell wall-associated fractions from wild-type *L. rhamnosus* GG and its derivative strain PA11 were analyzed by Western blot to visualize potential differences in the pilus ladder, typically ranging in the high molecular weight regions (Kankainen et al. 2009) (Fig. 3). In accordance with the immuno-EM observations (Fig. 1a), only pili of lower molecular weight seemed to be present on the cell surface of PA11, while the higher molecular weight pili seemed to be secreted into the culture medium (Fig. 3). This further supports the hypothesis that pili of mature length produced by PA11 are released in the medium because of the lack of sortase A activity, due to the mutation in the catalytic triad of SrtA. Work in *Staphylococcus aureus*, which like *L. rhamnosus* is a Gram-positive bacterium, showed the same geno-phenotype relationship, with abolishment of the Arg residue resulting in very strong, but not full, reduction of SrtA functionality (Marraffini et al. 2004).

### Analysis of the secreted proteome confirms increased secretion of pili by PA11

The proteome of secreted proteins of strain PA11 and wild-type *L. rhamnosus* GG was determined to investigate if the SrtA defect also affected other proteins beyond the SpaCBA pili. Secretome analysis resulted in 27 proteins that were enriched in the supernatant of the PA11 strain compared to the parental strain (Table 1; Supplementary File 2). Of interest, seven of these stood out as they made up the majority (over 75%) of the peptide counts. All of these secreted proteins harbored an LPXTG-motif and thus normally relied on SrtA activity for their anchoring to the cell surface (indicated in Table 1) (Douillard et al. 2014). The most abundant peptide counts derived from the large adhesin exoprotein of unknown function (LGG_RS13990) and the lactocepin protein (LGG_RS13070), a putative protease that in *L. casei* was shown to have potent immunomodulatory properties (von Schillde et al. 2012). In conclusion, the secretion of sortase-dependent proteins due to the defect in SrtA in the PA11 strain, further illustrates the importance of SrtA in anchoring LPXTG-harboring proteins on the cell surface.

**Table 1:**
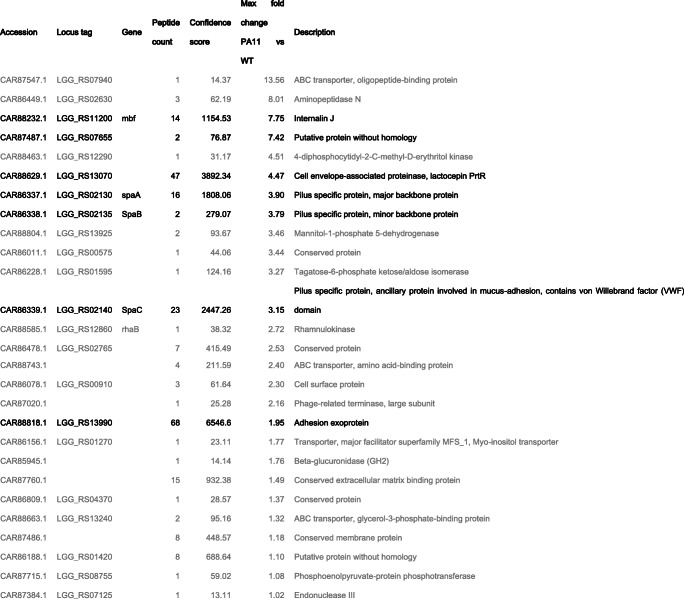
Proteome analysis of supernatant of *L. rhamnosus* GG PA11 derivative. Proteins enriched in the PA11 derivative are listed below. Sortase-dependent proteins are depicted in black. Full overview in Supplementary File 2

## Discussion

Here, we report the isolation and characterization of strain PA11, a food-grade derivative of the model probiotic *L. rhamnosus* GG that is known for its strong mucosal adherence due to the mucus-binding capacity of SpaC, which is the decorating and cap pilin of the SpaCBA pili (Kankainen et al. 2009). Using a combination of genomics and proteomics, immune EM visualization, and a set of functional analyses, we showed that strain PA11 has a mutation in house-keeping sortase A (*srtA)* gene, is deficient in mucus binding, and secretes its SpaCBA pili and other sortase-dependent proteins in the environment.

Sortase A is known to be involved in the covalent anchoring of the newly synthesized pili to lipid II structures in peptidoglycan (Marraffini et al. 2006; Clancy et al. 2010; Jacobitz et al. 2017). The mutation in the *srtA* gene of strain PA11 (nucleotide change at position 632 from G → A) affects amino acid residue 211 and results in an amino acid change from a positively charged Arg into a neutral Gln (Supplementary File 1, Fig. 2). Alignment of the *L. rhamnosus* GG sortase A (CAR88038.1) to other members of the highly conserved sortase A family cd06165, revealed that the affected Arg residue is part of the strongly conserved catalytic triad of the sortase A family: His, Cys (part of the TLXTC catalytic domain), and Arg (Fig. 2) (Marraffini et al. 2004). The conserved Arg residue at position 211 is essential for catalysis. It functions as a base, facilitating thiolate formation during sortase-mediated cleavage and transpeptidation, thus stabilizing the key substrate-enzyme intermediate required for efficient catalysis. Disruption of this Arg in *Staphylococcus aureus* severely reduced, but not completely eliminated, sortase A function, by almost completely abolishing transpeptidation (Marraffini et al. 2004). Given the strong conservation and essentiality of this Arg residue in SrtA efficacy (Fig. 2), its disruption explains the genotypic basis for the strain PA11 phenotype (Fig. 1). Indeed, strain PA11 is characterized by release of mature pili in the direct environment of the cells, with only a small fraction potentially still being attached to the cell surface (Fig. 1a). This is in accordance with reports in *S. aureus* (Marraffini et al. 2004) and might point towards the underlying mechanism of SrtA being unable to anchor the SpaCBA pili to lipid II. Abolishment of the housekeeping sortase SrtA has been shown to affect proper attachment of pili and by extension of other sortase-dependent proteins to the cell wall also in other Gram-positive bacteria, like *Corynebacterium diptheriae* (Swaminathan et al. 2007), *Lactobacillus plantarum* WCFS1 (Remus et al. 2013), *Lactobacillus acidophilus* NCFM, and *Lactobacillus gasseri* (Call et al. 2015). In these studies, it was reported that the failure to retain these sortase-dependent proteins to the cell surface often results in the loss of important strain phenotypes, like immunomodulation or adhesion (Remus et al. 2013; Call et al. 2015). These observations corroborate our findings further and support our conclusion that the mutation resulting in the Arg211Gln substitution in the housekeeping SrtA enzyme results in secretion of mature pili in the environment and ultimately in a strong reduced mucus-binding capacity of strain PA11. It will be interesting to validate this further using a site-directed mutagenesis approach in future studies although this may be technically challenging as *L. rhamnosus* GG is not so amenable to genetic changes as other model strains like *Lactococcus lactis*. Hence, in a previous work, we used *Lactococcus lactis* expressing *L. rhamnosus* GG pili for studying the effect of the sortase genes (both *srtA* and *srtC1*) and showed that removal or functional disruption of the housekeeping sortase resulted in a similar phenotype in this heterologous host (Douillard et al. 2014).

Microscopic analysis using pili-directed antibodies as a means to visualize SpaCBA pili and their components, resulted in the confirmation that PA11 secretes its pili in the environment, while only a small fraction of its pili remains bound to the cell envelope. Further Western blot analysis illustrated that these are low molecular weight pili and that the high molecular weight pilin agglomerates are secreted in the milieu surrounding PA11 cells. Retention of these low molecular weight pili could be caused by very low residual sortase A activity, but more likely options include the linkage of pili oligomers by hydrophobic interactions to the bacterial envelope or weak sortase C activity linking SpaB to lipid II, as has been earlier suggested (Douillard et al. 2014). Overall, the lack of high molecular weight pili in PA11 supports our finding that PA11 performs poorly in mucus-binding assays (with a more than 6-fold reduction) compared to its parental *L. rhamnosus* GG strain (wild type, Fig. 1d). This confirms that the SpaCBA pili are the main molecules involved in the high mucus adhesion phenotype of *L. rhamnosus* GG (Kankainen et al. 2009). The low residual mucus binding of strain PA11 may be due to the presence of some low molecular weight pili with the mucus-binding SpaC on its cell envelope, which is in accordance with our Immuno EM observations (Fig. 1). Apart from the SpaCBA pili, there are also other adhesins present on the cell surface of *L. rhamnosus* GG, which play a minor role in mucus binding, like the MBF protein, which is also a sortase-dependent protein (von Ossowski et al. 2011).

The use *L. rhamnosus* PA11 may have biotechnological advantages as it allows relative cost-effective and efficient enrichment of sortase-dependent proteins from the supernatant. This is extremely challenging to achieve using wild-type *L. rhamnosus* GG given the covalent association of its sortase-dependent proteins to the cell surface. The SpaCBA pili in particular have been implicated as the key modulators of *L. rhamnosus* GG extraordinary adhesion capacity, but also have a strong therapeutic potential. Earlier work has identified them as immunomodulatory molecules (Lebeer et al. 2012; Tytgat et al. 2016b), and they can be used in efforts to combat the important nosocomial pathogen Vancomycin-resistant enterococci (VRE) (Tytgat et al. 2016a).

In total, we found seven sortase-dependent proteins to be enriched in the secretome of PA11. Based on theoretical predictions, there is genomic potential of 19 sortase-dependent proteins present in *L. rhamnosus* GG (Douillard et al. 2014). However, we know from previous research that not all of these are actually expressed by *L. rhamnosus* strains, e.g., SpaFED pili (Reunanen et al. 2012). Interestingly, among the secreted sortase-dependent proteins in the PA11 strains are several proteins with therapeutic potential. The generated strain PA11 can thus be used to study the therapeutic potential of other sortase-dependent proteins with an immunomodulatory potential. These include the lactocepin protein (LGG_RS13070), a putative protease also known as PrtP, that is found in related Lactobacilli and in *L. casei* and that was shown to have potent immunomodulatory properties (von Schillde et al. 2012). Another abundant and large (2603 amino acids) secreted protein is the adhesion exoprotein (LGG_RS13990), a Leucin repeat protein that is partly conserved in Lactobacilli but presently has no assigned function and hence needs further functional characterization.

In conclusion, the here-characterized *L. rhamnosus* GG–derived PA11 strain opens novel avenues towards the exploration of these key molecules, both in a fundamental and therapeutic setting. Further enhancing the potential of strain PA11 is its non-GMO nature, rendering both the strain and its products safe for use in humans given the current regulations (EU 2001).

## Supplementary information

Supplementary File 1 SNP tables from shotgun and single-molecule genome sequencing of PA11 (XLSX 33 kb)

Supplementary File 2 Proteomics of the secreted protein fraction of the PA11 strain compared to wild-type *L. rhamnosus* GG (XLSX 71 kb)

## Data Availability

The genome sequence of *L. rhamnosus* GG PA11 can be found in the NCBI Sequence Read Archive (SRA) database with accession number SAMN04440354, and all sequencing data was deposited as part of BioProject PRJNA309744. The proteomics data is included in supplementary material. *L. rhamnosus* PA11 has been deposited at the DSM-Z Braunschweig, Germany and is available under number DSM 111733.
